# Protease Cleavage Leads to Formation of Mature Trimer Interface in HIV-1 Capsid

**DOI:** 10.1371/journal.ppat.1002886

**Published:** 2012-08-23

**Authors:** Xin Meng, Gongpu Zhao, Ernest Yufenyuy, Danxia Ke, Jiying Ning, Maria DeLucia, Jinwoo Ahn, Angela M. Gronenborn, Christopher Aiken, Peijun Zhang

**Affiliations:** 1 Department of Structural Biology, University of Pittsburgh School of Medicine, Pittsburgh, Pennsylvania, United States of America; 2 Department of Pathology, Microbiology and Immunology, Vanderbilt University School of Medicine, Nashville, Tennessee, United States of America; The Salk Institute for Biological Studies, United States of America

## Abstract

During retrovirus particle maturation, the assembled Gag polyprotein is cleaved by the viral protease into matrix (MA), capsid (CA), and nucleocapsid (NC) proteins. To form the mature viral capsid, CA rearranges, resulting in a lattice composed of hexameric and pentameric CA units. Recent structural studies of assembled HIV-1 CA revealed several inter-subunit interfaces in the capsid lattice, including a three-fold interhexamer interface that is critical for proper capsid stability. Although a general architecture of immature particles has been provided by cryo-electron tomographic studies, the structural details of the immature particle and the maturation pathway remain unknown. Here, we used cryo-electron microscopy (cryoEM) to determine the structure of tubular assemblies of the HIV-1 CA-SP1-NC protein. Relative to the mature assembled CA structure, we observed a marked conformational difference in the position of the CA-CTD relative to the NTD in the CA-SP1-NC assembly, involving the flexible hinge connecting the two domains. This difference was verified via engineered disulfide crosslinking, revealing that inter-hexamer contacts, in particular those at the pseudo three-fold axis, are altered in the CA-SP1-NC assemblies compared to the CA assemblies. Results from crosslinking analyses of mature and immature HIV-1 particles containing the same Cys substitutions in the Gag protein are consistent with these findings. We further show that cleavage of preassembled CA-SP1-NC by HIV-1 protease *in vitro* leads to release of SP1 and NC without disassembly of the lattice. Collectively, our results indicate that the proteolytic cleavage of Gag leads to a structural reorganization of the polypeptide and creates the three-fold interhexamer interface, important for the formation of infectious HIV-1 particles.

## Author Summary

HIV-1 virions assemble as immature particles that must undergo proteolytic maturation to become infectious. During the maturation process, the Gag polyprotein is cleaved into matrix (MA), capsid (CA), nucleocapsid (NC), and p6 proteins, and CA assembles to form a mature viral capsid. Here, we determined the structures of CA and CA-SP1-NC assemblies using cryo-electron microscopy, which revealed a marked conformational difference at the CA C-terminal domain in CA-SP1-NC compared to CA. We demonstrated that formation of mature interhexamer contacts critically depends on cleavage of the CA-SP1-NC and a resulting conformational change in CA. Our results provide new insights into the mechanism of HIV-1 maturation and are valuable for developing new inhibitors that target the interhexamer interface to block HIV-1 maturation.

## Introduction

The Gag polyprotein encompasses the major structural elements responsible for assembly of retroviruses, including human immunodeficiency virus type 1 (HIV-1). Assembly and budding involve Gag self-association and interactions with the host cell plasma membrane, the viral genomic RNA, and host cell dependency factors, leading to the formation of immature, non-infectious, spherical virus particles [1], [2]. The immature particles undergo maturation, resulting in a dramatic morphological rearrangement of viral components [3], [4]. This process is initiated by proteolytic cleavage of Gag into the structural proteins, matrix (MA), capsid (CA), and nucleocapsid (NC), as well as additional peptide sequences that vary among retroviruses (p6 and spacer peptides, SP1 and SP2, in the case of HIV-1) [5]. In the mature virion, MA (N-terminal Gag) remains associated with the viral membrane, and CA condenses into a distinct conical capsid shell (core) that encapsulates the viral enzymes reverse transcriptase and integrase along with the viral RNA genome, which is coated with NC [6].

The structures and functions of mature MA, CA, and NC proteins and of the mature capsid have been studied extensively through biochemical [7]–[12], biophysical [13]–[17] and structural analyses [12], [17]–[28], while the structures of immature Gag and the maturation intermediates are less well known. The overall architecture of the immature HIV-1 Gag and Gag-cleavage mutants have been studied using cryo-electron tomography (cryoET) [1], [2], [29]–[32], revealing partial coverage of the inner viral membrane by Gag, as a hexagonal lattice with ∼80 Å spacing [1], [30], [31]. The roles of specific HIV-1 Gag components in immature particle assembly [2] have been analyzed, and mutational analyses revealed that dimerization of the CA portion of Gag is critical for efficient assembly [11]. The NC component of Gag, which binds the viral RNA, aids in Gag-Gag interactions and particle assembly [29], [33]. Further, *in vitro* assembly of Gag, and of the CA-SP1-NC Gag fragment, depends upon NC interactions with nucleic acid [34], [35].

The precise mechanisms underlying transition from the “immature" Gag lattice to the mature capsid lattice, and the conformational changes that take place during maturation, are not well understood. Several models have been proposed, including de novo assembly of CA monomers or hexamers after proteolysis [22] and trigger-mediated conformational switch mechanisms [36], [37]. Mutations and drugs that block cleavage at CA-SP1 and SP1-NC prevent core condensation and result in impaired infectivity [38], [39]. Therefore, structural information on the C-terminal portion of Gag and the conformational changes that are associated with CA-SP1-NC cleavage and release of SP1 and NC will aid in our understanding of the maturation process.

To study the intersubunit interfaces contributing to the immature HIV-1 lattice, we used cryoEM and real-space helical reconstruction of *in vitro* assembled HIV-1 CA-SP1-NC tubes and obtained density maps at 13 Å resolution; we compared these with those of HIV-1 CA tubes at 11 Å resolution. Molecular docking of atomic CA structures into both density maps revealed a distinct conformational difference in the CA-CTD: namely a 34° rotation of the CTD relative to the NTD via the connecting hinge. Our data support the model in which proteolytic cleavage of Gag results in a structural reorganization of the inter-hexamer (trimer) interface that is required for mature capsid formation.

## Results/Discussion

### CryoEM structures of the CA-SP1-NC and CA assemblies

Previous studies on the mature capsid structure revealed several intermolecular interfaces that are critical for capsid function, namely the NTD-NTD, NTD-CTD, CTD dimer and CTD trimer interfaces [12], [17]–[28]. We sought to determine whether the equivalent interfaces exist during an intermediate stage of virus maturation and to delineate the structural changes that occur upon proteolytic release of CA from Gag. In particular, we assembled CA-SP1-NC tubes in the presence of nucleic acid [40] and compared the ‘immature’ CA structure in these assemblies with that of ‘mature’ CA tubes by CryoEM.

**Figure 1:**
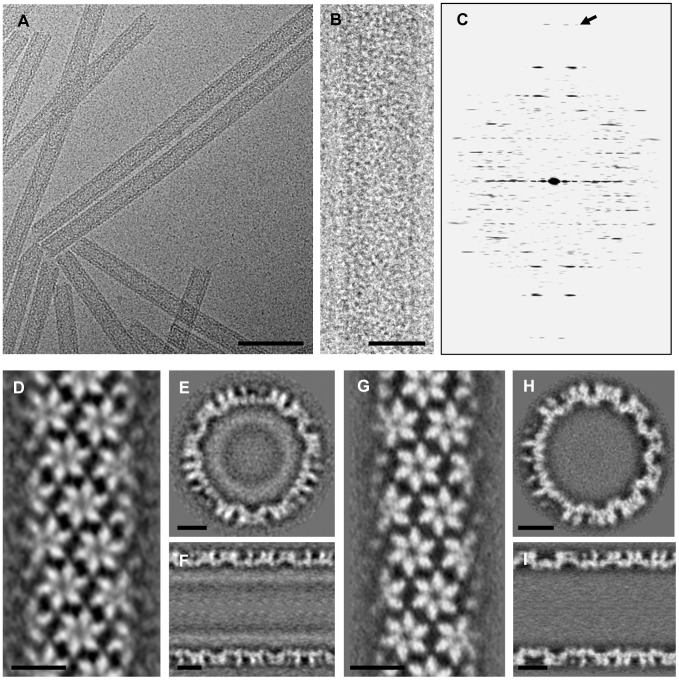
CryoEM and image reconstruction of HIV-1 CA-SP1-NC and CA tubular assemblies. (A–B) CryoEM micrographs of recombinant HIV-1 CA-SP1-NC assembled into tubes. (C) The computed Fourier transform of the tube shown in B. The arrow points to the layer-line at 23 Å resolution. (D–F) Final density map of CA-SP1-NC tubes from the (−14, 11) helical family, displayed as three orthogonal slices: parallel to the tube axis and close to the surface (D), perpendicular to the tube axis (E), and parallel to and through the tube axis (F). (G–I) The final density map of CA tubes from the (−12, 11) helical family, displayed as in (D–F). Scale bars, 100 nm in (A), 25 nm in (B), and, 10 nm in (D–H).

The CA-SP1-NC tubes exhibited double-layer densities (Fig. 1A & B), with an average diameter of 468±10 Å for the outer layer and 270±8 Å for the inner layer. Even though the diameter between the tubes varied, the distance between the outer and inner layer densities was invariant with a value of 100±2 Å. The CA-SP1-NC tubes displayed well-ordered helical symmetry and exhibited layer lines to at least 23 Å resolution (Fig. 1C). Among many helical families, we selected six of the best quality tubes of (−14, 11) helicity and carried out real-space helical reconstruction [41]. A final 3D density map at 13 Å resolution was obtained (Fig. S1A). For comparison, we also processed twelve CA tubes, each possessing (−12, 11) helical symmetry, to generate a density map at 11 Å resolution (Fig. 1G–I and Fig. S1C).

The CA-SP1-NC tubular structure exhibits two-fold symmetry, with an overall two-fold phase residual of 27.8°. The dimensions of the surface unit cell are a = 95.1 Å, b = 102.6 Å, γ = 109.7° (measured at radius = 234 Å), slightly smaller than those obtained from the CA (−13, 11) tubes analyzed previously, at 16 Å resolution [20], and the CA (−12, 11) tubes described here (a = 99 Å, b = 102 Å, γ = 108° (measured at radius = 213 Å)), at 11 Å resolution. The CA-SP1-NC density map displays a 74 Å thick outer layer with a hexagonal surface lattice and a 53 Å thick, more diffuse, inner layer (Fig. 1D–F). The outer layer consists of CA hexamers (Fig. 1D), similar to CA assemblies, while the inner layer most likely comprises the NC protein and nucleic acid. The latter are required for CA-SP1-NC assembly. The poorly-defined densities in the inner layer suggest that the molecular structure in this region is flexible and/or poorly ordered, consistent with data from tomographic studies of immature particles [1] that revealed the absence of an ordered lattice in the NC region. There is a distinctive space (∼36 Å) between the CA and NC density layers, suggesting that the SP1 peptide may span this distance. The CA reconstruction at 11 Å resolution from the assembly with (−12, 11) helical symmetry (Fig. 1G–I), exhibits essentially the same CA structure as that described previously for the (−13, 11) helical assembly [20]. For comparison with CA-SP1-NC, the density map from (−12, 11) helical family tubes was used for subsequent structural analysis, owing to its higher resolution.

### Molecular docking of a pseudo-atomic structure into the CA-SP1-NC density map

**Figure 2:**
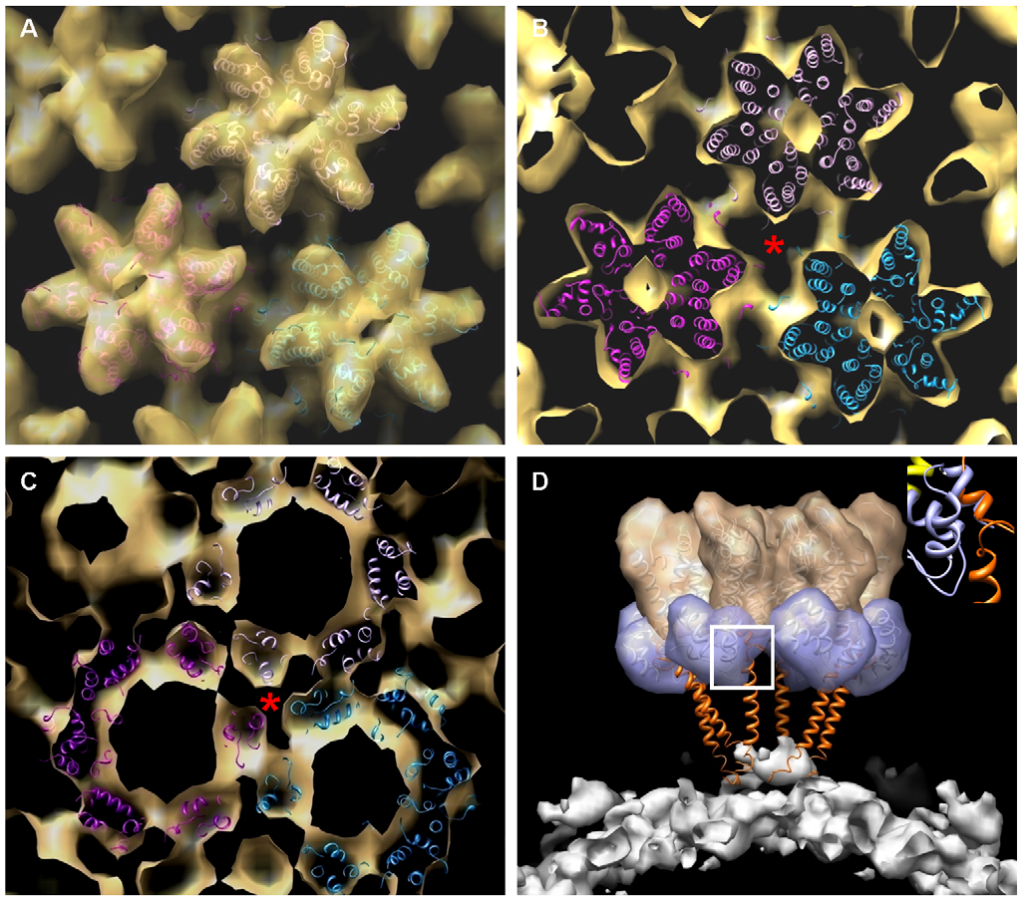
Molecular docking of the CA-SP1-NC density map. (A–C) Docking of the CA-NTD (PDB 3h47) and CA-CTD (PDB 2kod) domain models, independently, into the CA region (gold) of the density map contoured at 1.22σ, enclosing 100% volume. Shown are a view onto the tube surface (A) and slab views to highlight the NTD (B) and CTD regions (C). Three neighbouring CA hexamer models are in cyan, magenta and pink. The red star indicates the pseudo-three fold axis where the trimer interface is found in the CA assembly [20]. (D) Placing of the SP1 segment (PDB 1u57) into the CA-SP1-NC tubular density map. The CA-NTD in gold, CTD in light blue and SP1 in orange are shown for a single hexamer in the assembly. The density contour at the NC/DNA region is shown in white. The inset (top right), magnified from the white box, shows the overlapped region of the CA-CTD and SP1 models.

For a detailed comparison of the CA structure in the CA and CA-SP1-NC assemblies, we docked atomic models of the CA-NTD (PDB 3h47) [19] and the solution CA-CTD dimer (PDB 2kod) [20], separately, into the CA-SP1-NC density map using a correlation-based automated rigid-body fitting method [20]. The pseudo-atomic model fits the CA-SP1-NC density envelope very well (Fig. 2), as measured by cross-correlation function (CCF) between the model and density map (CCF = 0.95 for CA-NTD and 0.94 for CA-CTD, respectively). Three of the previously characterized CA intermolecular interfaces, NTD-NTD, NTD-CTD, and CTD-CTD dimer [19]–[21], are also observed in the CA-SP1-NC tubular structure, although details of the contact residues may vary. Interactions between neighboring CA-SP1-NC hexamers are primarily mediated by the CTD dimers (Fig. 2A & B)); however, in contrast to the previous CA structure [20] and the current 11 Å map, the intermolecular trimer contact at the pseudo three-fold axis is not observed (Fig. 2C).

Unlike CA, modeling of SP1 and NC in the density map was not straightforward. The distinctive pillar-like structures previously described in immature VLPs [1], [22], [30] are not observed, and only very weak electron density is present in the region between the CA layer and the inner NC/nucleic acid layer of the CA-SP1-NC assemblies (Fig. 1E & F). Further, the diffuse density in the NC/nucleic acid region precludes the docking of a NC structural model with any confidence and suggests that, in these tubes, NC and the nucleic acid are not well-ordered, consistent with previous studies [1]. This notion is further supported by the lower resolution in this region (24 Å compared to 13 Å in the CA region (Fig. S1A & B)). With regard to the very weak electron density in the SP1 region, we considered several possible scenarios: 1) SP1 may form an α-helical bundle [42], [43] that does not possess helical symmetry, thus the density would not be apparent with helical reconstruction; 2) SP1 has a flexible connection to the CA-CTD, thus the density for SP1 is smeared out after averaging; and 3) SP1 adopts a random coil structure with some nascent, fluctuating helical population, as suggested by the NMR analysis of the CA-CTD-SP1-NC protein in solution [44]. Based on the present structure, which was reconstructed using helical symmetry, we are unable to distinguish between these scenarios. As a first approximation, we constructed a model for CA-SP1 by connecting the SP1 NMR structure model (PDB 1u57), determined in the presence of trifluoroethanol [45], to the CA-CTD by superimposing the overlap region (LEEMMTACQG) from the CA-CTD (PDB 2kod) and the SP1 structures (Fig. 2D, inset). Interestingly, the resulting six α-helical SP1 segments point toward the interior diffuse NC/nucleic acid density (Fig. 2D). Thus, it is plausible that SP1 adopts α-helical conformation in the assembled state, as recently suggested [46].

### Structural reorganization in HIV-1 capsid assembly upon proteolytic cleavage

**Figure 3:**
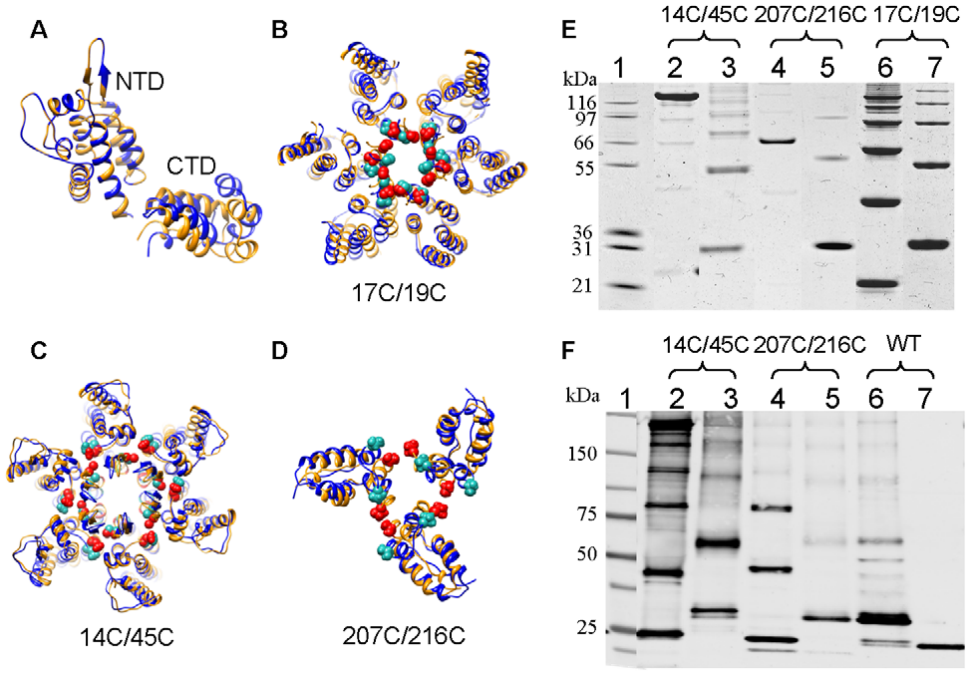
Comparison of the intermolecular interfaces and chemical crosslinking patterns between CA and CA-SP1-NC structures. (A) Superposition of the CA monomer model from the assembled CA structure (gold) and that from the assembled CA-SP1-NC structure (blue). (B–D) Superposition of the CA-NTD models at the NTD interfaces (B&C) and CA-CTD models at the trimer interface (D). The color scheme is identical to (A). Space-filling representation highlights the specific residue pairs; 17/19 in (B), 14/45 in (C) and 207/216 in (D), with red corresponding to those from CA and cyan to those from CA-SP1-NC. (E) Non-reducing SDS-PAGE analysis of *in vitro* disulfide crosslinked CA (lanes 2, 4 and 6) and CA-SP1-NC assemblies (lanes 3, 5 and 7), stained with Coomassie Blue. Lanes were rearranged from original gels shown in Fig. S3. (F) Non-reducing SDS-PAGE analysis of crosslinking of mature (lanes 2, 4 and 7) and cleavage-defective HIV-1 CA6 particles (lanes 3, 5 and 6). Proteins were detected by immunoblotting with a CA-specific antibody.

The pseudo-atomic models for assembled CA and CA-SP1-NC tubes permits delineation of the structural differences in CA when C-terminal Gag sequences are present. Within the CA monomer, a major conformational difference is observed in the relative orientation between the CTD and the NTD (Fig. 3A). Specifically, the CTD domain is rotated approximately 34 degrees relative to the NTD through a flexible hinge (Video S1). Our current data suggest that this hinge plays a pivotal role in transmitting CA conformational changes upon protease cleavage, further underscoring the importance of the hinge region in the formation of mature capsid [47]. To evaluate whether and how this change in the monomer impacts the interfaces in the assembly, we carried out detailed comparative analyses of the intermolecular NTD-NTD and CTD-CTD trimer interfaces in the assembled ‘mature’ and ‘immature’ capsids [20], [21]. While the first interface is involved in forming the CA hexamer or pentamer [18], [19], the latter connects CTD domains from adjacent hexamers and is important for forming the extended lattice [20].

The hexamer-forming NTD-NTD interactions present in the assembled CA are largely retained in the CA-SP1-NC assembly, although some minor variations exist. The P17/T19 pair [10] (Fig. 3B), located at the center of the 18-helix barrel, is important in the hexamer arrangement [19]–[21], [25] and displays similar average Cα-Cα distances, namely 6.3 and 8.0 Å in CA and CA-SP1-NC, respectively (Table S1). In contrast, the A14/E45 pair [10], located half way into the barrel (Fig. 3C), exhibits an average Cα-Cα distance difference of 3.9 Å between the two structures and noticeable variability among pairs within the CA-SP1-NC structure. Specifically, while the six A14/E45 pairs in the CA structure exhibit uniform Cα-Cα distances of 8.1±0.2 Å, in the CA-SP1-NC structure, four of six pairs display an average Cα-Cα distance of 13.0±0.7 Å, with the other two pairs being significantly closer (∼10 Å apart).

The most dramatic difference in the CA-SP1-NC assembly compared to that of CA is found at the critical CTD trimer interface that we previously characterized [20]. The P207/T216 Cα-Cα distance at this interface is about 9 Å in the CA structure, while in the CA-SP1-NC structure these residues are separated by nearly 20 Å (Fig. 3D, Table S1). In fact, no electron density was observed at the pseudo three-fold axis in the CA-SP1-NC map (Fig. 2C), in stark contrast to the CA density map [20]. Therefore, it appears that a major conformational change takes place after proteolytic cleavage at the CA-SP1 and/or SP1-NC junctions, leading to formation of the unique trimer interface in the mature capsid.

### Probing structural alterations during HIV-1 maturation by chemical crosslinking

To test our structural models and verify the predicted intermolecular contacts at interfaces in CA and CA-SP1-NC assemblies, double cysteine mutations were introduced at P17/T19, A14/E45, and P207/T216 for chemical crosslinking. The CA and CA-SP1-NC mutants were all competent for assembly (Fig. S2), and the crosslinking pattern of each mutant agreed very well with our structural model predictions (Figs. 3E and S3). After crosslinking at 17C/19C, the CA [10] and CA-SP1-NC assemblies displayed a similar ladder of oligomers, suggesting that the associated interface is unperturbed upon proteolytic cleavage. In contrast, crosslinking of 14C/45C resulted in predominant hexamers in the CA assembly [10] but was much less efficient in CA-SP1-NC, with the dimeric product representing the major crosslinked species. This pattern is consistent with our structural model in which two (of the six) pairs are close enough for disulfide crosslinking in the CA-SP1-NC assembly (Table S1). An even more pronounced difference between the two assemblies was observed after crosslinking at the CTD trimer interface (Fig. 3E lanes 4 & 5); no significant accumulation of trimer was observed in CA-SP1-NC, consistent with the altered spatial arrangement at the trimer interface.

To extend the above *in vitro* studies to HIV-1 viral particles, we examined the behavior of the identical cysteine mutants in mature virions and compared it with particles in which the specific cleavage sites between CA and NC were abolished (CA6) [39]. To evaluate spontaneous disulfide crosslinking, particles were recovered from transfected cells and analyzed by non-reducing SDS-PAGE and immunoblotting with a CA-specific antiserum. The resulting data are in agreement with the *in vitro* analysis (Fig. 3F). In particular, the CA protein in mature 14C/45C virions was readily crosslinked into hexamers (lane 2), while in the corresponding CA6 mutant particles, the protein accumulated predominantly in the dimer state (lane 3). Further, in mature particles, the 207C/216C spontaneous crosslink resulted in dimer and trimer forms of CA (lane 4) that were not observed in CA6 particles (lane 5). Taken together, our crosslinking studies support the cryoEM-derived structural model and suggest that the trimer interface in the mature HIV-1 capsid is formed only after protease cleavage of Gag.

### Implications for HIV-1 capsid maturation: structural reorganization without disassembly

**Figure 4:**
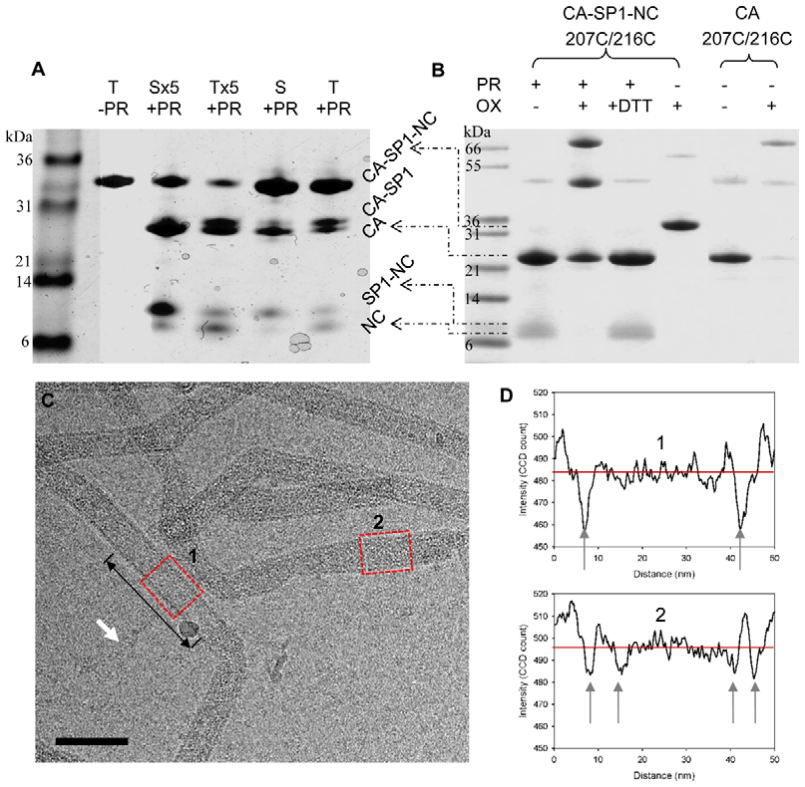
Protease cleavage of CA-SP1-NC assemblies. (A) SDS-PAGE analysis of HIV-1 protease (PR)-treated, unassembled soluble CA-SP1-NC (S) and CA-SP1-NC tubular assemblies (T), visualized by Coomassie Blue staining. The cleavage products, CA-SP1, CA, SP1-NC, and NC are labeled. Samples treated with 5 times more protease were marked as Sx5 and Tx5. (B) Non-reducing SDS-PAGE analysis of crosslinked, protease-treated CA-SP1-NC 207C/216C assembly. (C) CryoEM image of protease treated CA-SP1-NC tubular assemblies. A segment, indicated by the double-headed arrow, of single-layer tube, similar to CA tubes, was observed. The white arrow indicates linear densities observed only in protease treated samples. Scale bar, 100 nm. (D) Cross-sectional density profiles of the single-layer tube (box 1) and double-layer tube (box 2) marked in C. Gray arrows point to the high density regions in the projection image.

To mimic the process of HIV-1 maturation *in vitro*, we performed HIV-1 protease digestion studies of preassembled CA-SP1-NC tubes and identified distinct patterns of digestion, compared to the unassembled protein. HIV-1 protease cleavage of unassembled CA-SP1-NC occurred primarily at the CA-SP1 site, yielding CA and SP1-NC as products, with very little NC (Fig. 4A). However, in assembled CA-SP1-NC, the efficiency of cleavage at this site was greatly reduced, possibly because SP1 is less flexible in the assembled lattice [44]. Instead, cleavage between SP1 and NC was more efficient in assembled complexes, similar to Gag processing in immature particles [48]. At this juncture the question arose whether the mature CA-CTD trimer interface is formed upon protease processing of CA-SP1-NC tubes. Remarkably, the protease-cleaved CA-SP1-NC 207C/216C assemblies were able to form crosslinked CA dimers and trimers similar to the mature CA 207C/216C assemblies (Fig. 4B), indicating that the trimer contacts form upon protease cleavage. In addition, the *in vitro* proteolysis process appears to involve concerted conformational changes, as we consistently observed a stretch of single-layered tube, apparently digested and of lighter intensity, flanked by the undigested, double-layered CA-SP1-NC tubes in the cryoEM images (Fig. 4C & D). The linear densities (Fig. 4C, white arrows) likely correspond to the released NC/DNA fragments, which were only observed in protease-treated samples but not in untreated samples. The fact that we observed non-random and local proteolysis implies that the maturation process through the release of SP1-NC likely involves reorganization of CA interfaces, rather than disassembly and reassembly of CA subunits. This, does not exclude the possibility of partial or complete dissociation of immature lattice during early maturation steps, as suggested by the recent structure of an immature Gag assembly lattice of M-PMV [49]. Considering that there are two fractions of CA in HIV-1 particles, one of which assembles into the mature capsid [30], [50], and that fewer than one-half of Gag protein is expected to be bound to the viral genomic RNA [51], we hypothesize that the fraction of RNA-bound Gag is selected for capsid assembly and remains a part of the lattice. By contrast, in our CA-SP1-NC assembly every protein molecule is bound to nucleic acid, and CA may remain associated with the complex following protease cleavage.

**Figure 5:**
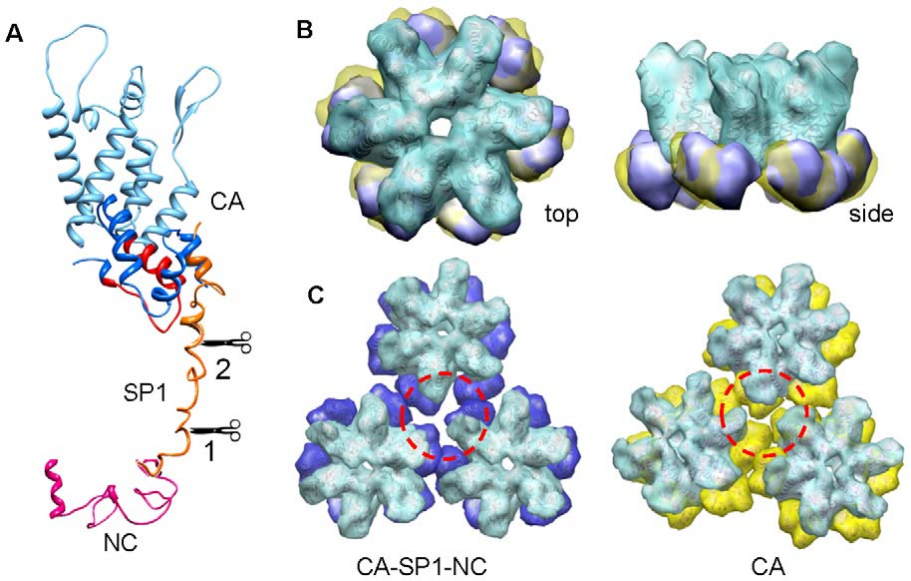
A model depicting formation of the mature capsid trimer interface upon protease cleavage. (A) Structure model of the CA-SP1-NC protein was composed using the CA model derived from molecular docking (see Fig. 2), combined with a SP1 partial helix (hypothetical) and the NC model (PDB 1f6u). The linker between CA-CTD and SP1 is in close contact with the MHR region of CA-CTD (in red). Scissors point to the two protease cleavage sites, Site 1 and Site 2, respectively. (B) Superposition of the CA hexameric structural models from CA-SP1-NC (blue) and CA structures (yellow) viewed from top and side. The two CA models were aligned at the CA-NTD region (light blue). (C) Comparison of the trimer interfaces (red-dashed circles) between CA-SP1-NC (blue) and CA structures (yellow).

Based on our findings, we propose a working model for the mechanism of HIV-1 capsid maturation with regard to the C-terminus of Gag (Fig. 5): in immature assemblies, initially only the SP1-NC cleavage site is readily accessible to the HIV-1 protease (Site 1). This results in NC and viral RNA release, as well as disorder in the SP1 segment. This, in turn, permits access of protease to the CA-SP1 site (Site 2), causing cleavage at that site. Removal of NC/RNA and SP1 not only destabilizes the immature lattice [52], but also allows the CA-CTD to reorient relative to the NTD, creating new contacts along the trimer interface and forming the mature capsid contacts [20] (Fig. 5B&C). Whether proteolysis between SP1-NC allows formation of the trimer interface or whether release of SP1, by CA-SP1 cleavage, is also needed requires further investigation.

The order of Gag cleavage by protease is known to follow the sequence SP1-NC>MA-CA≥SP2-p6>NC-SP2>CA-SP1 [48]. The question, therefore, arises: does the conformation of CA-CTD in the CA-SP1-NC structure represent a relevant structural intermediate, given that NC is released before MA and p6? We suggest the answer is yes, for the following reasons: 1) p6 release is most likely irrelevant to the CA-CTD conformation [53], [54]; and 2) high-resolution structures show that the MA and CA-NTD domains are connected by a flexible linker and that MA cleavage does not appear to affect the CA-CTD structure, but results in formation of a beta-hairpin at the N-terminus of CA-NTD [55], [56]. The importance of the MA-CA cleavage for the immature-to-mature lattice conversion has been implicated previously [36], [55], [57], [58], but role of the β-hairpin in HIV-1 maturation is not clearly established. The N-terminus of CA-NTD in the CA-SP1-NC construct may fold in a mature-like configuration, as indicated by the similarity of CA-NTD structures and crosslinking pattern of 17C/19C in both CA-SP1-NC and mature CA tubes. This notwithstanding, the CA-CTD in fact shows a major difference between CA-SP1-NC and CA tubes, and clearly is in an “immature-like" configuration, exemplified by the CTD trimer interface and supported by *in vivo* and *in vitro* disulfide crosslinking experiments. Therefore, the CA conformation in the CA-SP1-NC construct may represent a maturation intermediate, with only the CA-NTD on the path to maturation.

Our data establish that release of SP1-NC is essential for formation of the trimer interface present in the mature viral capsid. This interface controls mature capsid structure: mutations that prevent cleavage of CA-SP1 result in unstable, incompletely formed capsids [39]. Furthermore, mutations in residues at the trimer interface, including Q219A, K203A, and E213A/Q, alter the intrinsic stability of the viral capsid and impair HIV-1 infectivity [7], [20] with no apparent effect on capsid structure. Thus, the intersubunit trimer interface controls both capsid structure as well as stability. CA-SP1 is also the target of HIV-1 maturation inhibitors, including BVM and PF96, which act by inhibiting cleavage of this site [59]–[61]. BVM, in particular, has been reported to stabilize the immature capsid lattice, suggesting that cleavage of SP1 results in conversion from “immature-intermediate" to the mature capsid structure [62]. It should be noted that CA-SP1-NC represents an intermediate conformation that is different from the initial immature state before any cleavage. Consistent with this, a Gag mutant defective for CA-SP1 cleavage fails to display a clear immature lattice [62]. While the present work indicates that the trimer contacts critically depend on cleavage of CA-SP1-NC, a detailed understanding of HIV-1 maturation awaits a detailed structural description of this region in the immature HIV-1 capsid lattice; structural knowledge on this interface will be important for developing small molecules that target the interface to inhibit maturation.

## Materials and Methods

### CA and CA-SP1-NC assembly

Recombinant A92E and double cysteine mutant HIV-1 CA and CA-SP1-NC proteins were expressed and purified as previously described [20], [27]. CA tubes were assembled at 80 µM concentration in 1 M NaCl and 50 mM Tris-HCl (pH 8.0) at 37°C for 1 hour. CA-SP1-NC tubes were assembled at 300 µM concentration with 60 µM TG50 (IDT, Coralville, IA) in 250 µM NaCl, 50 mM Tris-HCl (pH 8.0) buffer at 4°C for 19 hours.

### CryoEM specimen preparation and data collection

CA and CA-SP1-NC assemblies (2 µl) were applied to the carbon side of a glow discharged perforated Quantifoil grid (Quantifoil Micro Tools, Jena, Germany). 2.5 µl dilution buffer (0.25 M NaCl, 50 mM Tris-HCl pH 8.0) was added to the back side of the grid, which was then blotted with a filter paper and plunge-frozen in liquid ethane using a home-made manual gravity plunger. Low dose (10∼15 e^−^/Å^2^) projection images were collected on Kodak SO-163 films with an FEI Tecnai TF20 electron microscope at a nominal magnification of 50,000 and underfocus values ranging from 1.0 to 2.5 µm. The best micrographs were digitized using a Nikon super coolscan 9000 ED scanner (Nikon, Japan) at a resolution of 4000 dpi.

### Three-dimensional reconstruction

Well-ordered long tubes were Fourier transformed and indexed for helical symmetry. Six CA-SP1-NC tubes belonging to the helical family (−14, 11) and twelve CA tubes belonging to the helical family (−12, 11) were included in the final reconstruction of CA and CA-SP1-NC density maps respectively. Image processing and 3D reconstruction were carried out as previously described [41]. The structures of CA and NC were further refined separately using helical refinement programs [63]. During the refinement, helical symmetry and contrast transfer function correction were applied. The resulting density maps were visualized with Chimera [64]. The resolutions of the final 3D reconstructions were estimated from the Fourier shell correlation (FSC) curve using the FSC-0.5 cut-off criterion.

### Rigid body fitting

Pseudo-atomic models of CA hexamers were constructed by docking the following NTD and CTD domain models (PDB 3h47 for CA-NTD and PDB 2kod for CA-CTD) into the density map separately. To obtain a model of HIV-1 CA hexamer, first, the fitting was performed with the tool “Fit in Map" implemented in Chimera [64]. Then the solution was refined with the program “colores" in Situs package [65]. The relative orientation of CA-CTD with respect to NTD in CA and CA-SP1-NC structures was measured in Chimera by structural comparison. A pseudo-atomic model for CA-SP1was constructed by aligning the overlapping segment (LEEMMTACQG) from both CA-CTD structure (PDB 2kod) and SP1 structure (PDB 1u57) using the tool “MatchMaker" in Chimera.

### Analysis of disulfide crosslinking in tubular assemblies and HIV-1 particles

Crosslinking analysis of *in vitro* assembled CA and CA-SP1-NC mutants was carried out as previously described [12]. Briefly, 30 µl P207C/T216C, P17C/T19C and A14C/E45C CA and CA-SP1-NC mutants were preassembled in the presence of 50 µM DTT under the conditions described above. The assembled material was then subjected to centrifugation at 20,000 g at room temperature in an Eppendorf centrifuge 5417R for 15 minutes. The pellet was oxidized with oxidization mix (60 µM CuSO_4_, 267 µM 1,10-Phenanthroline. Sigma) and immediately quenched with 20 mM iodoacetamide and 3.7 mM Neocuproine (Sigma). The reaction mix was electrophoresis on 4–20% polyacrylamide gradient gels (Bio-Rad, Hercules, CA) and stained with Coomassie-Blue.

Crosslinking analysis of pelleted HIV-1 particles was performed as previously described [20]. Virus particles were derived by transfection of the full-length HIV-1 proviral construct R9 and mutant derivatives. The CA-SP1-NC cleavage site mutant was derived from the pNL4-3-based construct CA6 [39] by transfer of a BssHII-ApaI fragment into R9. Particles were pelleted from the supernatants of transfected 293T cells, followed by lysis in non-reducing Laemmli buffer and electrophoresis on 4–20% polyacrylamide gradient Criterion gels (Bio-Rad). Proteins were electrophoretically blotted to nitrocellulose membranes and detected by probing with a CA-specific polyclonal antibody. Bands were revealed with an Odyssey imaging system after probing with IR dye-conjugated anti-rabbit antibody.

### HIV-1 Protease digestion of CA-SP1-NC assemblies

CA-SP1-NC assembly solution (300 uM) was centrifuged at 20,000×g for 20 minutes. The pellet was then resuspended in protease digestion buffer (100 mM NaAc, 100 mM NaCl, 1 mM EDTA, 1 mM DTT, pH 5.5) with a final protein concentration of 80 µM. The soluble CA-SP1-NC protein without assembly was diluted directly to 80 µM with protease digestion buffer. For protease digestion experiment (Fig. 4A), 0.09 µM and 0.45 µM (5×) HIV-1 protease (Sigma) were incubated with CA-SP1-NC tubes for 5 hours and the reaction mixtures were analyzed by cryoEM. HIV-1 protease (kind gift from Dr. Celia Schiffer at the University of Massachusetts) at 0.45 µM concentration was used for the trimer interface crosslinking experiment with CA-SP1-NC 207C/216C mutant following protease cleavage (Fig. 4B). The digestion reaction mixtures were incubated at 37°C for 5 hours and then subject to SDS-PAGE and cryoEM analysis. The reaction products were separated by NuPAGE Novex 4–12% Bis-Tris gel (Invitrogen) and visualized by Coomassie blue staining.

## Supporting Information

Figure S1 **Three-dimensional reconstruction and molecular docking of CA-SP1-NC assemblies.** (A–C) Fourier shell correlation (FSC) and phase residual plots of the CA-SP1-NC density map calculated from the CA region (A) and from the NC-DNA region (B), and of the CA density map (C). The resolutions of the maps are 13 Å at FSC = 0.5, or at a phase residue of 65° for CA-SP1-NC (A), and 11 Å at FSC = 0.5, or at a phase residue of 68° for CA (C).
(TIF)

Figure S2 **Representative negatively-stained EM images of the crosslinked CA and CA-SP1-NC assemblies.** (A–E) CA-SP1-NC wild-type and double-cysteine mutants. (F–J) CA wild-type and double-cysteine mutants.
(TIF)

Figure S3 **SDS-PAGE analysis of intermolecular crosslinking of** ***in vitro*** **assembled CA and CA-SP1-NC tubes with engineered cysteine pairs.** Each triplet contains non-oxidized (lanes 2, 5, 8, 11, 14, 17), oxidized (lanes 3, 6, 9, 12, 15, 18), and oxidized then reduced (lanes 4, 7, 10, 13, 16, 19) samples.
(TIF)

Video S1 **The movie shows the conformational changes at CTD between the mature CA assembly (gold) and ‘immature’ CA-SP1-NC assembly (Blue) in a hexamer, viewed from top.**
(AVI)

Video S2 **The movie shows the conformational changes at CTD between the mature CA assembly (gold) and ‘immature’ CA-SP1-NC assembly (Blue) in a hexamer, viewed from side.**
(AVI)
