# Supplementary figures and images for: Protease Cleavage Leads to Formation of Mature Trimer Interface in HIV-1 Capsid
Source: PLoS Pathog. 2012 Aug 23;8(8):e1002886. doi: 10.1371/journal.ppat.1002886 (PMC3426514; doi:10.1371/journal.ppat.1002886)

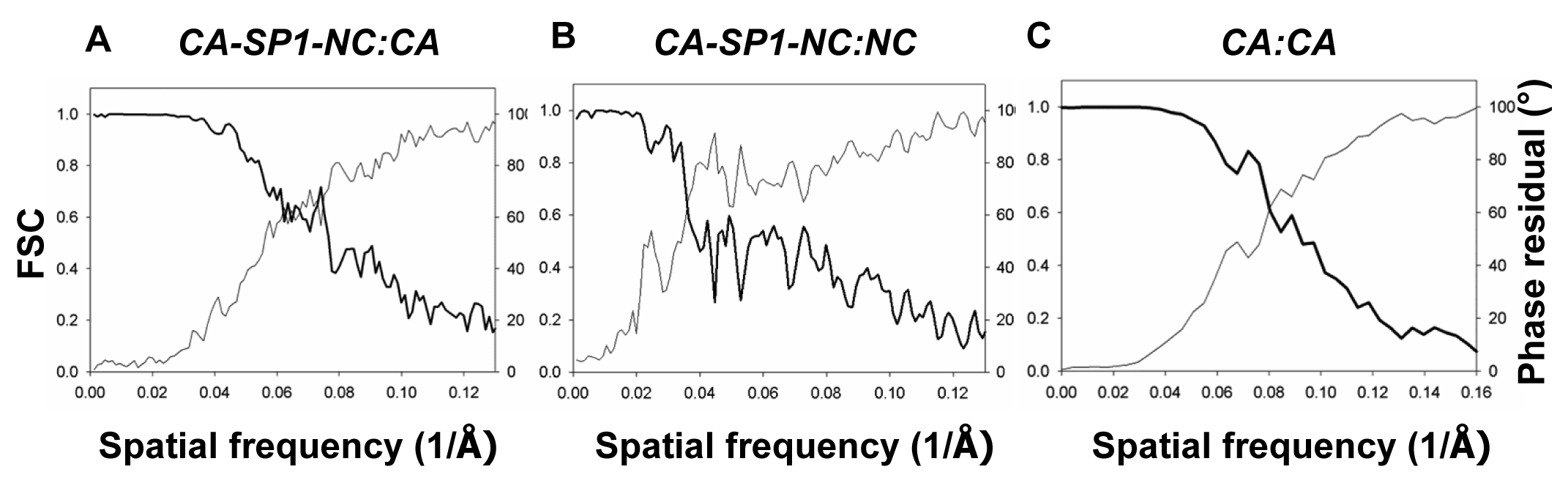

Supplement: Figure S1 — Three-dimensional reconstruction and molecular docking of CA-SP1-NC assemblies. (A–C) Fourier shell correlation (FSC) and phase residual plots of the CA-SP1-NC density map calculated from the CA region (A) and from the NC-DNA region (B), and of the CA density map (C). The resolutions of the maps are 13 Å at FSC = 0.5, or at a phase residue of 65° for CA-SP1-NC (A), and 11 Å at FSC = 0.5, or at a phase residue of 68° for CA (C). (TIF) [file ppat.1002886.s001.tif]

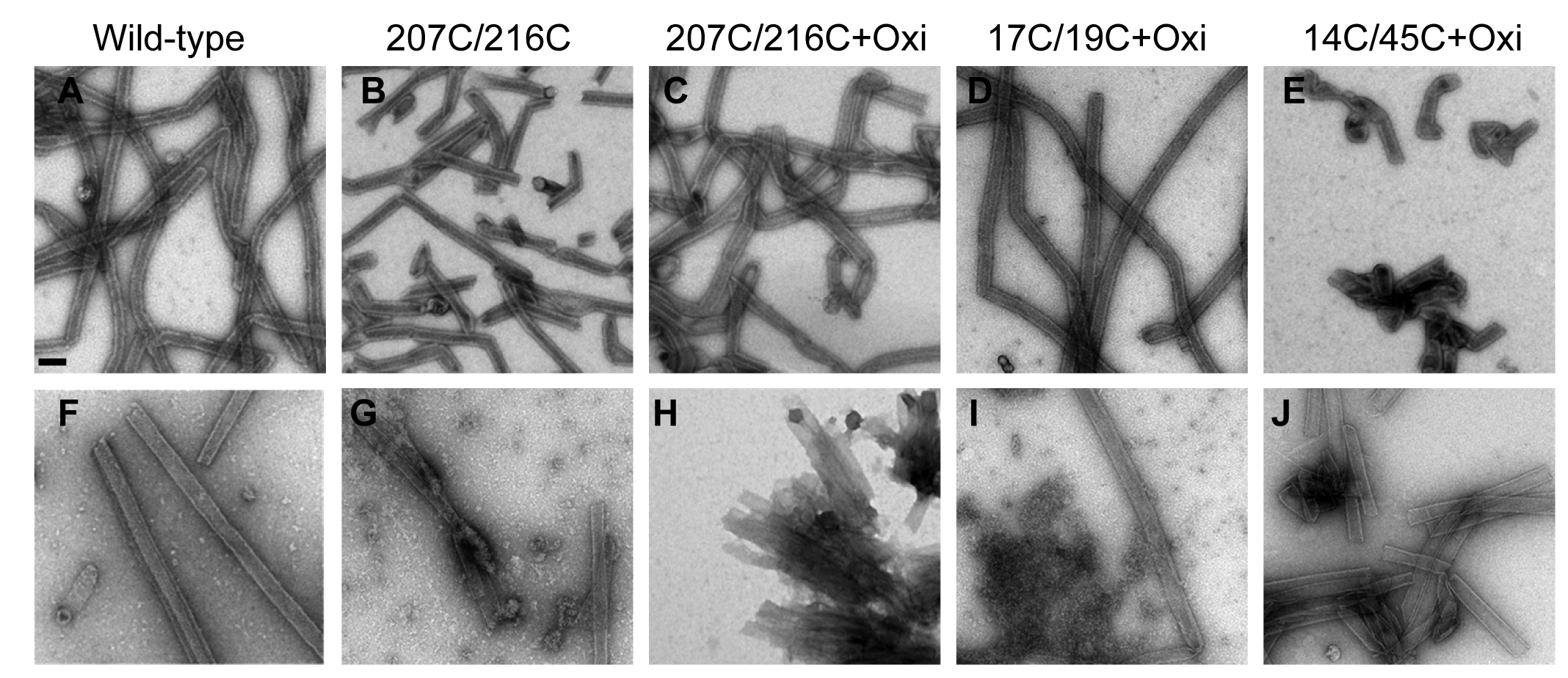

Supplement: Figure S2 — Representative negatively-stained EM images of the crosslinked CA and CA-SP1-NC assemblies. (A–E) CA-SP1-NC wild-type and double-cysteine mutants. (F–J) CA wild-type and double-cysteine mutants. (TIF) [file ppat.1002886.s002.tif]

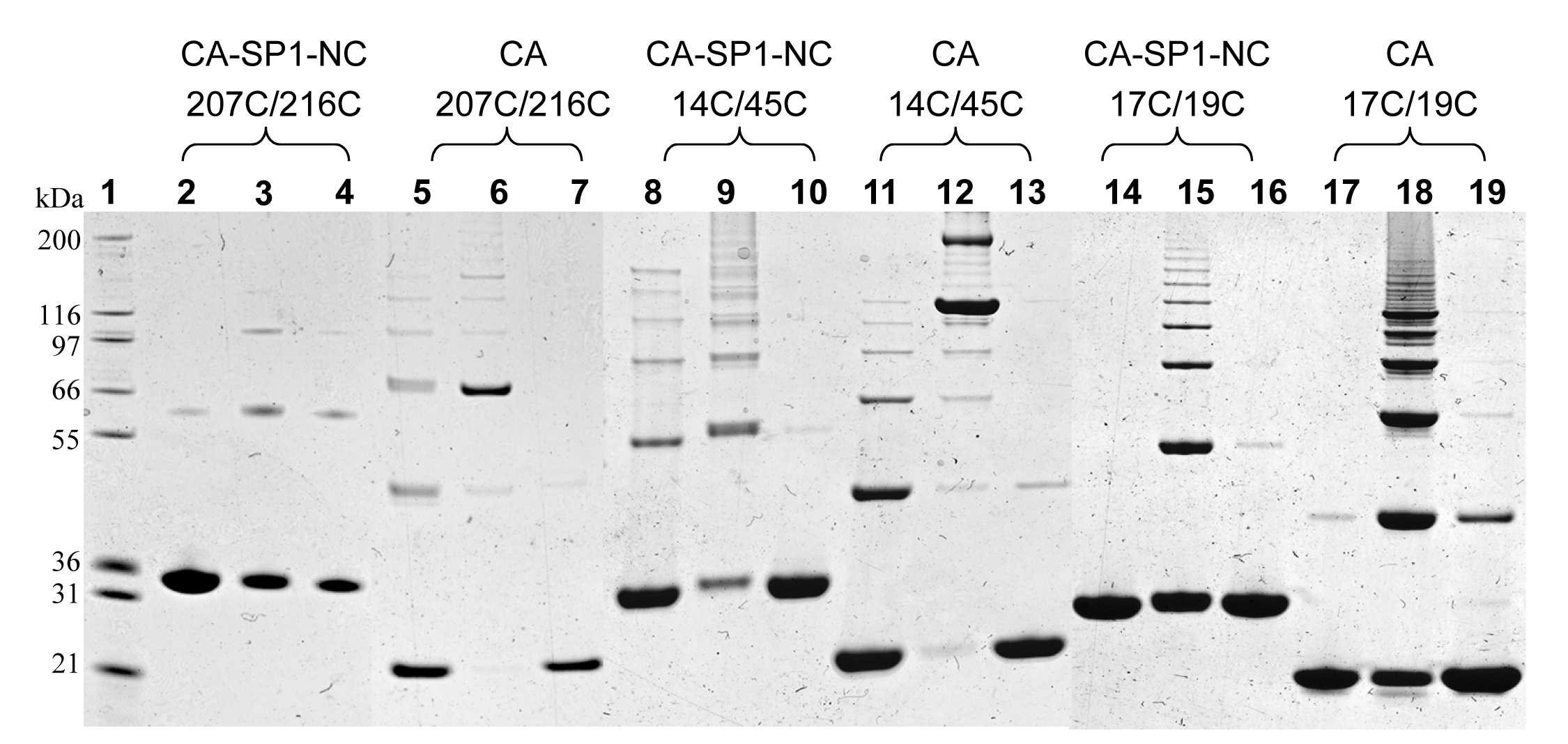

Supplement: Figure S3 — SDS-PAGE analysis of intermolecular crosslinking of in vitro assembled CA and CA-SP1-NC tubes with engineered cysteine pairs. Each triplet contains non-oxidized (lanes 2, 5, 8, 11, 14, 17), oxidized (lanes 3, 6, 9, 12, 15, 18), and oxidized then reduced (lanes 4, 7, 10, 13, 16, 19) samples. (TIF) [file ppat.1002886.s003.tif]
